# Adjuvant chemotherapy-associated lipid changes in breast cancer patients: A real-word retrospective analysis

**DOI:** 10.1097/MD.0000000000021498

**Published:** 2020-08-14

**Authors:** Tao He, Chengshi Wang, Qiuwen Tan, Zhu Wang, Jiayuan Li, Tao Chen, Kaijun Cui, Yunhao Wu, Jiani Sun, Danxi Zheng, Qing Lv, Jie Chen

**Affiliations:** a Department of Breast Surgery, West China School of Medicine/West China Hospital, Sichuan University; b Clinical Research Center for Breast Diseases, Laboratory of Molecular Diagnosis of Cancer, and Department of Medical Oncology, West China Hospital, Sichuan University; c Department of Breast Surgery, West China Hospital, Sichuan University; d Laboratory of Molecular Diagnosis of Cancer, West China Hospital, Sichuan University; e West China School of Public Health and West China Fourth Hospital, Sichuan University; f Department of Endocrinology and Metabolism, Adrenal Center, West China Hospital of Sichuan University; g Department of Cardiology, West China Hospital of Sichuan University; h West China School of Medicine/West China Hospital, Sichuan University, China.

**Keywords:** Adjuvant chemotherapy, Breast cancer, Dyslipidemia

## Abstract

Adjuvant chemotherapy may cause alterations in serum lipids in postoperative breast cancer (BC) patients, but the specific alterations caused by different chemotherapy regimens remain unclear. The aim of this study was to investigate the status of serum lipids pre- and post-chemotherapy and to compare the side effects of different chemotherapy regimens on serum lipid.

We retrospectively analysed the lipid profiles of 1934 consecutive postoperative BC patients who received one of the following chemotherapy regimens:

(1) doxorubicin and cyclophosphamide followed by paclitaxel (AC-T);
(2) epirubicin and cyclophosphamide followed by paclitaxel (EC-T);
(3) cyclophosphamide and paclitaxel (TC); and
(4) fluorouracil, cyclophosphamide, and epirubicin (FEC).

The levels of triglycerides (TG), total cholesterols (TC), and low-density lipoprotein (LDL-C) were significantly elevated in patients who received chemotherapy regimens above (*P* < .001). With respect to different chemotherapy regimens, FEC had less side effects on lipid profiles (TG (*P* *=* .006), high-density lipoprotein (HDL-C) (*P* < .001), and LDL-C (*P* < .001)) than TC regimen and AC-T and EC-T regimen. Also, the incidence of newly diagnosed dyslipidemia after chemotherapy was lower in FEC group than TC group and AC-T and EC-T group (*P* < .001). Additionally, the magnitude of the alterations in lipid profiles (TG, TC, HDL-C, and LDL-C) was greater in premenopausal patients than that of the postmenopausal patients (*P* *=* .004; *P* < .001; *P* *=* .002; *P* *=* .003, respectively). Moreover, after adjusting for multiple baseline covariates, anthracycline-plus-taxane-based regimens (AC-T and EC-T) were still statistically associated with a high level of TG (*P* *=* .004) and a low level of HDL-C (*P* *=* .033) after chemotherapy compared with FEC regimen. Also, body mass index (BMI) > 24 was associated with abnormal lipid profiles (TG, TC, HDL-C, LDL-C) post-chemotherapy compared with BMI ≤ 24 (*P* < .001; *P* *=* .036; *P* *=* .012; *P* *=* .048, respectively).

BC patients receiving chemotherapy may have elevated lipid profiles, and anthracycline-based regimen had less side effects on lipid profiles compared with regimens containing taxane. Therefore, it is necessary to take lipid metabolism into consideration when making chemotherapy decisions and dyslipidemia prevention and corresponding interventions are indispensable during the whole chemotherapy period.

## 1 Introduction

Breast cancer (BC) is one of the most common malignancies all over the world, with an incidence of approximately 2,100,000 new cases and a mortality rate of approximately 630,000 worldwide in 2018.^[1]^ However, the mortality rates for BC patients have declined in recent decades due to early diagnosis and comprehensive treatments, thus leading to more BC survivors.^[2–4]^

In recent years, it has been found that with the increasing survival rate of BC patients, some of them finally died due to non-cancer causes.^[5]^ Based on the latest available statistics, the proportion of non-cancer-related death has increased gradually, which has even exceeded cancer-related death in early BC survivors.^[6,7]^ Cardiovascular diseases (CVD), which accounts for 35% of non-cancer-related deaths, are the most common cause of non-cancer-related death and competes with BC as the leading cause of death in older patients with early BC.^[8]^

Adjuvant chemotherapy improves both disease-free and overall survival of BC survivors,^[9–11]^ but nowadays it's side effects gradually draw increasing attention. For instance, systemic agents such as anthracyclines might increase cardiac toxicity and contribute to CVD, especially in survivors who live longer.^[12,13]^ Moreover, previous epidemiological studies have indicated that dyslipidemia is closely related to CVD and that high levels of serum lipid profiles are likely to induce CVD.^[14]^ Therefore, adjuvant chemotherapy-associated dyslipidemia and CVD deserve urgent attention. It is worth noting that earlier studies reported that serum lipid profiles might increase after chemotherapy in BC patients.^[15]^ However, few studies have explored the side effects of different chemotherapy regimens on serum lipids to date.

Therefore, we retrospectively investigated the variations in serum lipid levels pre- and post-chemotherapy and explored the side effects of different chemotherapy regimens on lipid profiles.

## 2 Methods and materials

### 2.1 Patient selection

The medical records of all consecutive patients with BC were retrospectively collected from the Department of Breast Surgery, West China Hospital, Sichuan University between February 2009 and December 2016. Approval for this retrospective analysis from the Institutional Review Board and Ethics Committee of West China Hospital. And the need for consent from study participants was not required. Because this was a retrospective study, no human specimens are involved and the data was accessed anonymously. The inclusion criteria were as follows:

(1) female patients aged ≥18 years;
(2) patients surgically treated and pathologically diagnosed with BC;
(3) stage I to III BC;
(4) patients who received 1 of the following 4 common chemotherapy regimens: doxorubicin and cyclophosphamide followed by paclitaxel (AC-T), epirubicin and cyclophosphamide followed by paclitaxel (EC-T), cyclophosphamide and paclitaxel (TC), or fluorouracil, cyclophosphamide, and epirubicin (FEC); and
(5) patients who had adequate organ function and Eastern Cooperative Oncology Group (ECOG) ≤2.

The exclusion criteria were as follows:

(1) BC patients with other malignancies;
(2) patients who received prior chemotherapy within the last year;
(3) pregnant and lactating women;
(4) patients who had not completed the established chemotherapy regimens;
(5) patients with incomplete data; and
(6) patients taking drugs affecting serum lipids.

### 2.2 Data collection and evaluated parameters

All clinicopathological data were reviewed from medical records. The variables of interest included body mass index (BMI), age at diagnosis, menopausal status, tumor location, surgical procedures, tumor TNM (Tumor Node Metastasis) stage,^[16]^ and pre- and post-chemotherapy lipid values (blood samples were collected in the morning on an empty stomach within a week before the first cycle of chemotherapy treatment and within 2 weeks after the end of the last cycle of chemotherapy treatment). According to the Chinese guidelines for the management of dyslipidemia,^[17]^ the biochemical parameters (total cholesterols (TC), triglycerides (TG), high-density lipoprotein (HDL-C), low-density lipoprotein (LDL-C)) related to dyslipidemia were categorized using cut-off values as follows: TG 1.7 mmol/L, TC 5.20 mmol/L, HDL-C 1.0 mmol/L, and LDL-C 3.4 mmol/L. Dyslipidemia was considered if patients met at least 1 of the following criteria: TG ≥1.7 mmol/L, TC ≥5.2 mmol/L, LDL-C ≥3.4 mmol/L, or HDL-C ≤1.0 mmol/L. Since menopausal status is closely related to serum lipids, participants in this study were further stratified into 2 groups by menopausal status: premenopausal and postmenopausal. Participants were classified as postmenopausal if they were not pregnant, were aged over 40 years and had no menstruation for at least 12 months.^[18]^ Also, since BMI may influence serum lipids, we then stratified the participants into 2 groups: BMI ≤ 24 and BMI > 24.^[19]^ Body weight and height were assessed for the determination of BMI, which was calculated as the weight divided by the height squared (kg/m^2^).

### 2.3 Chemotherapy regimens

According to the guidelines of Chinese Society of Clinical Oncology (CSCO) breast cancer, the specific chemotherapy regimens are as follows:

(1) AC-T (doxorubicin 60 mg/m^2^ and cyclophosphamide 600 mg/m^2^ every 3 weeks for 4 cycles followed by docetaxel 100 mg/m^2^ every 3 weeks for 4 doses);
(2) EC-T (epirubicin 90 mg/m^2^ and cyclophosphamide 600 mg/m^2^ every 3 weeks for 4 cycles followed by docetaxel 100 mg/m^2^ every 3 weeks for 4 doses);
(3) TC – docetaxel plus cyclophosphamide (4 cycles of docetaxel 75 mg/m^2^ and cyclophosphamide 600 mg/m^2^ administered every 3 weeks);
(4) FEC – 5-fluorouracil plus epirubicin and cyclophosphamide (6 cycles of triweekly 5-fluorouracil 500 mg/m^2^, epirubicin 100 mg/m^2^, and cyclophosphamide 500 mg/m^2^).

AC-T and EC-T are anthracycline plus taxane based regimens; TC is taxane-based regimen; and FEC is anthracycline-based regimen.

### 2.4 Statistics analysis

All statistical analyses were performed using the Statistical Package for the Social Science (SPSS), version 22.0 for Windows (SPSS Inc, Chicago, IL). Quantitative results were expressed as the mean ± standard deviation (SD). A two-sided *P* value of less than .05 was considered statistically significant. A paired-sample *T* test was used to compare the lipid values pre- and post-chemotherapy. The magnitude of variations in lipid profiles between pre- and post-chemotherapy among the 3 types of chemotherapy regimens were calculated, and the difference value (DV) was calculated as the post-chemotherapy lipid value minus the pre-chemotherapy lipid value. One-way analysis of variance was used to assess significant differences in the magnitude of variations in lipid profiles among the 3 types of chemotherapy regimens. Similarly, significant differences in the magnitude of variations in lipid profiles between pre- and post-chemotherapy among the premenopausal and postmenopausal group were also assessed by one-way analysis of variance. Additionally, chi-square test was used to compare the incidence of post-chemotherapy dyslipidemia in patients with normal serum lipids before chemotherapy among 3 types of chemotherapy regimens. Similarly, the incidence of post-chemotherapy hyperlipidemia in patients with normal serum lipids before chemotherapy between pre- and postmenopausal group was also compared by chi-square test. Moreover, we incorporated the significant variables associated with post-chemotherapy abnormal lipid profiles, which were identified by univariate analysis (*P* < .05), into a logistic regression model to identify the independent factors that were correlated with abnormal lipid profiles after chemotherapy.

## 3 Results

### 3.1 Patients’ characteristics

A total of 1934 eligible and consecutive BC patients were identified for this study. The median age of the 1934 BC patients was 48 years old (IQR 42–56). Among these people, 483 patients received AC-T regimen, 484 patients received EC-T regimen, 467 patients received TC regimen, and 500 patients received FEC regimen. As for menopausal status, 1184 were premenopausal and 750 were postmenopausal. With respect to the clinicopathological differences among the individuals in the 3 types of chemotherapy groups, for the premenopausal patients, significant differences in age, TNM stage, and molecular subtyping were observed (*P* < .05). There was no statistical significance in terms of BMI, smoking, tumor location, preoperative hypertension, preoperative dyslipidemia, or surgical procedures among the 3 chemotherapy groups (*P* > .05). For the postmenopausal patients, there were significant differences in age, preoperative hypertension, TNM stage, and molecular subtyping (*P* < .05). There was no significant difference in terms of BMI, smoking, preoperative dyslipidemia, surgical procedures, or tumor location among the 3 chemotherapy groups (*P* > .05). The clinicopathological characteristics are summarized in Table 1.

**Table 1:**
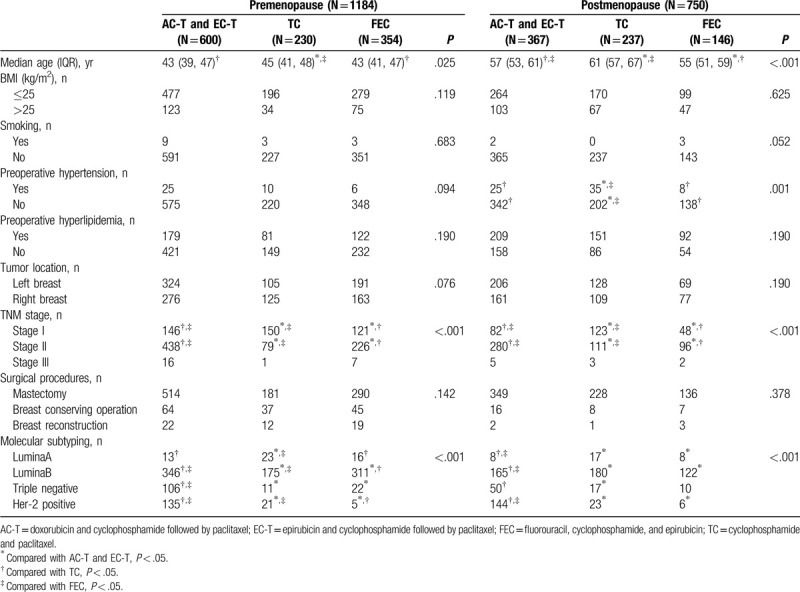
Demographic and clinical characteristics of the study population (n = 1934).

### 3.2 Serum lipid levels between pre- and post-chemotherapy

**Figure 1:**
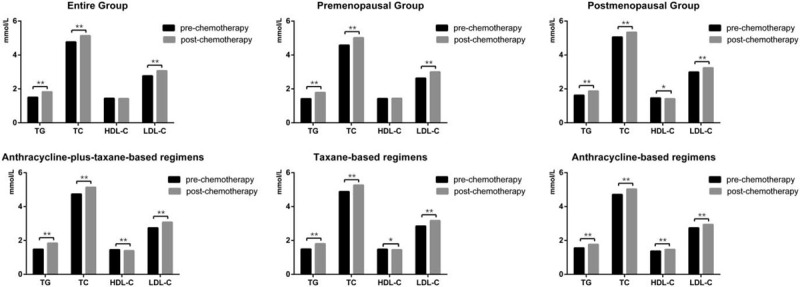
Serum lipid profiles between pre- and post-chemotherapy. ∗*P* < .05 for comparison of pre- vs post-chemotherapy. ∗∗*P* < .001 for comparison of pre- vs post-chemotherapy.

The effects of chemotherapy on serum lipid levels were shown in Table 2 and Figure 1. In this study, there were 1264 patients with elevated TG, 1299 patients with elevated TC, 1296 patients with elevated LDL-C, and 963 patients with decreased HDL-C after chemotherapy. For the entire group, we found a significant increase in TG (*P* < .001), TC (*P* < .001), and LDL-C (*P* < .001) after chemotherapy. However, in terms of HDL-C, there was no significant difference between pre- and post-chemotherapy (*P* = .368).

**Table 2:**
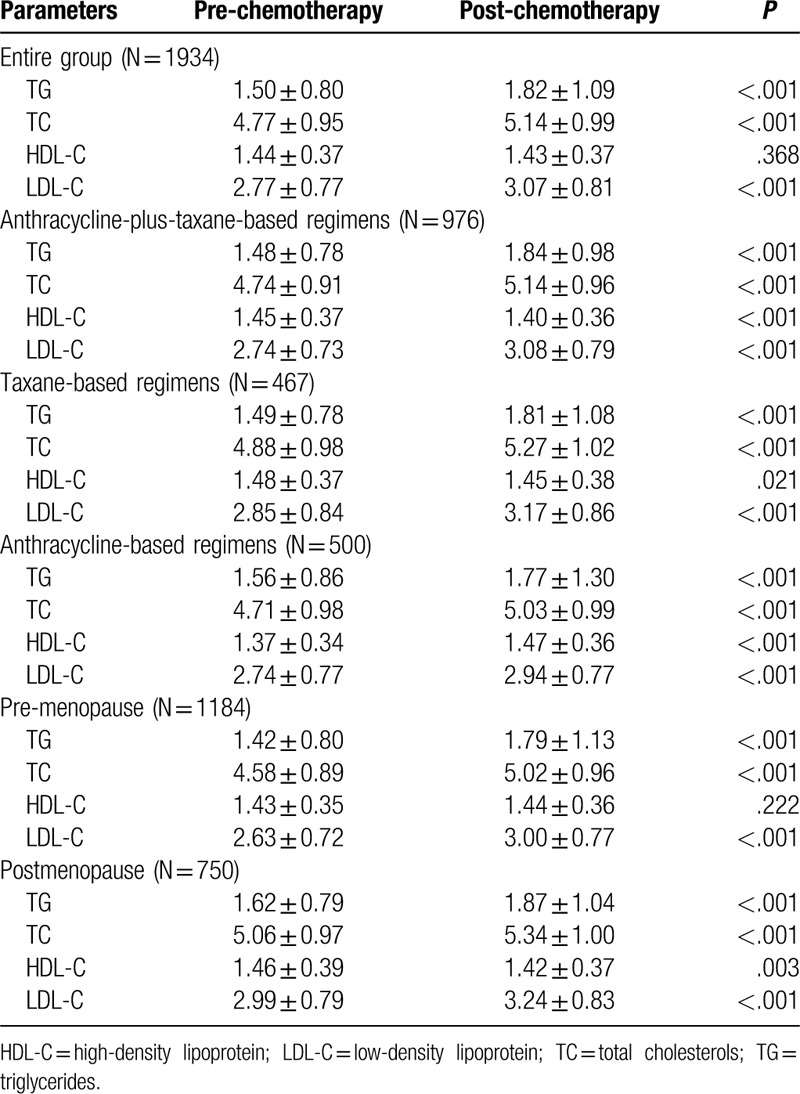
Comparison of the lipid profiles pre- and post-chemotherapy.

To assess chemotherapy regimens on serum lipids, the participants were divided into 3 groups: anthracycline-plus-taxane-based regimens group, taxane-based regimens group, and anthracycline-based regimens group. In terms of the patients received anthracycline-plus-taxane-based regimens, we found a significant increase in TG (*P* < .001), TC (*P* < .001), and LDL-C (*P* < .001); while a significant decrease in HDL-C (*P* < .001) after chemotherapy. Similarly, for patients received taxane-based regimens, a significant increase in TG (*P* < .001), TC (*P* < .001), LDL-C (*P* < .001), and a significant decrease in HDL-C (*P* = .021) were also observed after chemotherapy. In addition, for patients received anthracycline-based regimens, we found that TG (*P* < .001), TC (*P* < .001), HDL-C (*P* < .001), and LDL-C (*P* < .001) were all elevated after chemotherapy.

Since menopausal status is closely related to serum lipids, the study participants were then stratified into 2 groups by menopausal status: premenopausal and postmenopausal. For the premenopausal group, we found an increase in TG (*P* < .001), TC (*P* < .001), and LDL-C (*P* < .001) levels after chemotherapy. For the postmenopausal group, an increase was also noted in the levels of TG (*P* < .001), TC (*P* < .001), and LDL-C (*P* < .001) after chemotherapy; while the levels of HDL-C (*P* *=* .003) were decreased after chemotherapy.

### 3.3 Comparison of the magnitude of lipid alterations after chemotherapy

**Figure 2:**
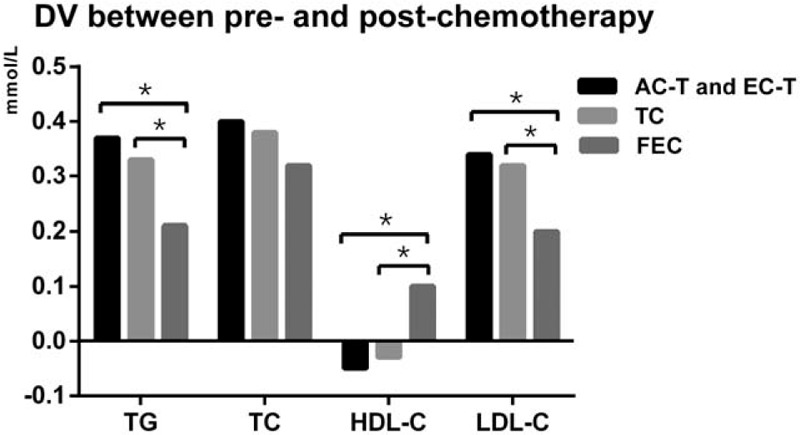
Compare the DV of serum lipid levels between post- and pre-chemotherapy in AC-T and EC-T group, TC group, and FEC group (DV = lipid value of post-operation minus lipid value of pre-chemotherapy). ∗*P* < .05 for comparison of DV among C-T and EC-T group, TC group, and FEC group. DV: difference value.

To explore the magnitude of lipid alterations in different chemotherapy regimens, we carried out further analysis. The data can be seen in Table 3 and Figure 2. For the alterations of TG levels, the magnitude of TG increase in the FEC group was less than the other 2 groups (*P* *=* .006). Similarly, the magnitude of LDL-C increase in the FEC group was also less than the other 2 groups (*P* < .001). In terms of HDL-C levels, HDL-C increased in patients who received FEC chemotherapy; while HDL-C decreased in the other 2 groups (*P* < .001). Additionally, there was no significant difference in the magnitude of the alterations of TC in all the 3 types of chemotherapy regimens (*P* *=* .21). In a word, FEC regimen may have less side effects on serum lipids compared with TC, AC-T, and EC-T regimens, especially in TG, LDL-C, and HDL-C levels.

**Table 3:**
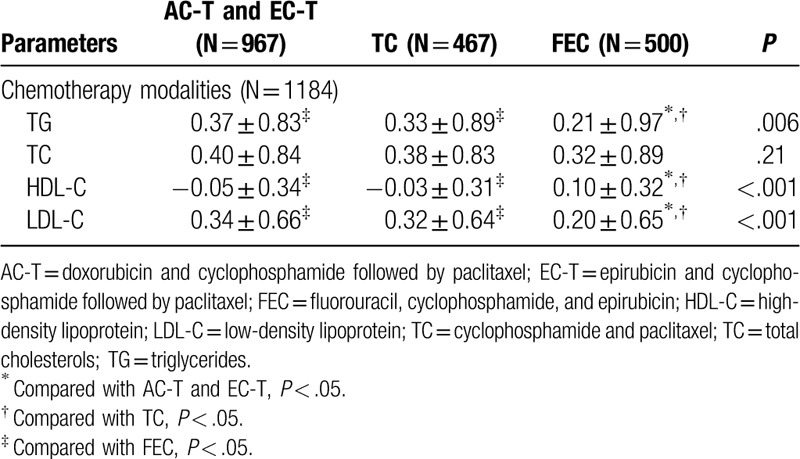
Comparison of magnitude of lipid profiles between pre- and post-chemotherapy among 3 types of chemotherapy regimens.

**Figure 3:**
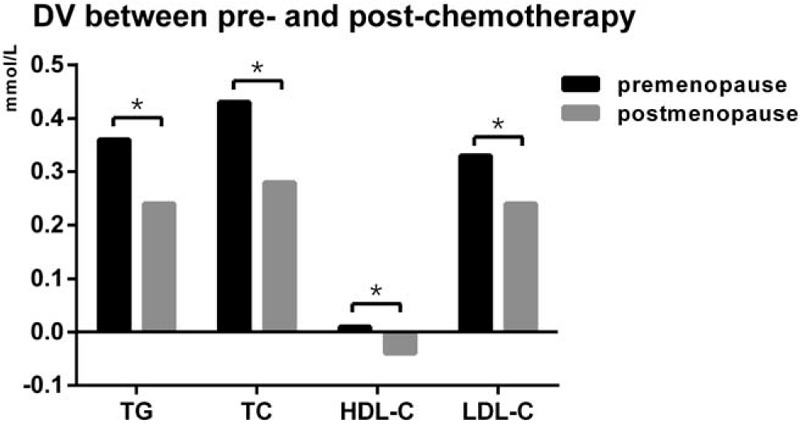
Compare the DV of serum lipid levels between post- and pre-chemotherapy in premenopausal group and postmenopausal group (DV = lipid value of post-operation minus lipid value of pre-chemotherapy). ∗*P* < .05 for comparison of DV between premenopausal group and postmenopausal group. DV: difference value.

In addition, we also compared the magnitude of lipid alterations between premenopausal and postmenopausal patients (Table 4 and Fig. 3). As for the alterations of TG, TC, and LDL-C levels, the magnitudes of the increase in TG, TC, and LDL-C were all greater in premenopausal patients than that of the postmenopausal patients (*P* *=* .004; *P* < .001; *P* *=* .003, respectively). As for the alterations of HDL-C levels, HDL-C increased in the premenopausal patients while the HDL-C decreased in the postmenopausal patients (*P* *=* .002). In general, the side effects of chemotherapy on serum lipid may be more remarkable in premenopausal patients compared with postmenopausal patients, especially in TG, TC, and LDL-C levels.

**Table 4:**
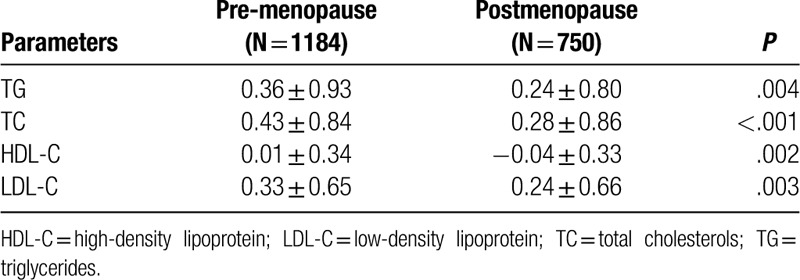
Comparison of magnitude of lipid profiles pre- and post-chemotherapy between pre- and postmenopausal BC patients.

**Figure 4:**
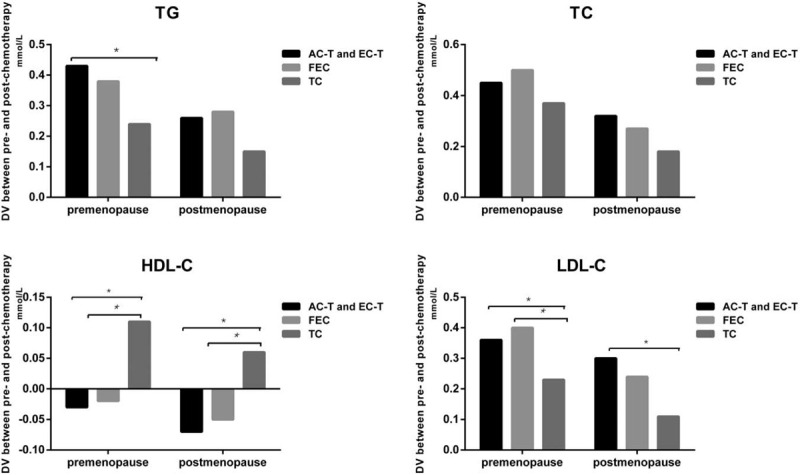
Compare the DV of serum lipid levels between post- and pre-chemotherapy in AC-T and EC-T group, TC group, and FEC group in pre- and postmenopausal group, respectively (DV = lipid value of post-operation minus lipid value of pre-chemotherapy). ∗*P* < .05 for comparison of DV among C-T and EC-T group, TC group, and FEC group. DV: difference value.

Since menopausal status and chemotherapy regimens both had different side effects on serum lipids, we carried out further layered analysis (Table 5 and Fig. 4). Firstly, we stratified all the participants into 2 groups: premenopausal and postmenopausal. For the premenopausal group, the magnitude of TG increase in the FEC group was less than that in the AC-T and EC-T group (*P* *=* .007). For LDL-C, the magnitude of LDL-C increase was also lower in the FEC group than that in TC and AC-T and EC-T groups (*P* = .002). For HDL-C, we found a decrease in both AC-T and EC-T group and TC group; and an increase in the FEC group (*P* < .001). For TC, there was no significant difference in the magnitude of TC alterations in the 3 types of chemotherapy regimens (*P* = .187). For the postmenopausal group, there was no significant difference in the magnitude of variations in either TG or TC levels among the 3 chemotherapy regimens (*P* = .265; *P* = .232, respectively). For HDL-C, we found an increase in the FEC group and a decrease in the other 2 groups (*P* < .001). For LDL-C, the magnitude of LDL-C increase was lower in the FEC group than that in the AC-T and EC-T group (*P* = .012). Generally, for postmenopausal patients, FEC regimen had less side effects on HDL-C and LDL-C levels than the other 2 regimens. For premenopausal patients, apart from HDL-C and LDL-C, FEC regimen also had less side effects on TG levels compared with the other 2 regimens.

**Table 5:**
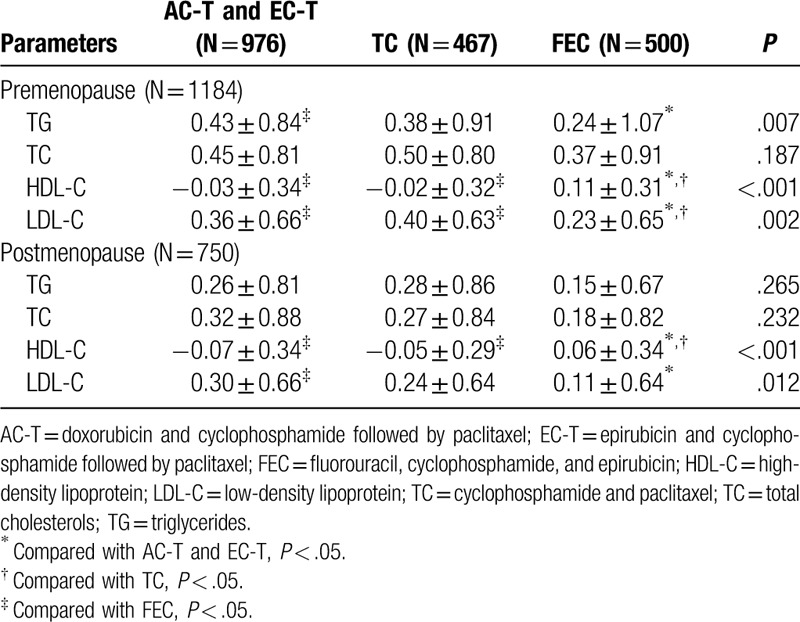
Comparison of change values of blood lipid between pre- and post-chemotherapy among 4 different chemotherapy modalities.

### 3.4 Incidence of newly diagnosed hyperlipidemia after chemotherapy

**Figure 5:**
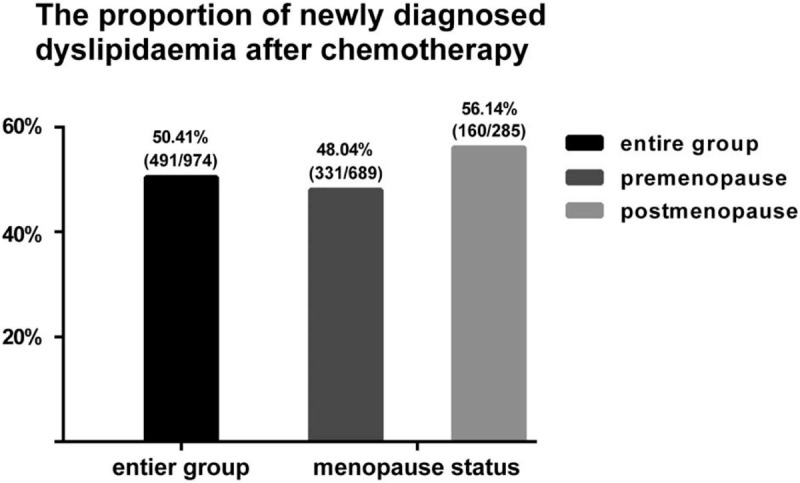
The percentage of newly diagnosed dyslipidemia after chemotherapy in the entire group, premenopausal group, and postmenopausal group.

**Figure 6:**
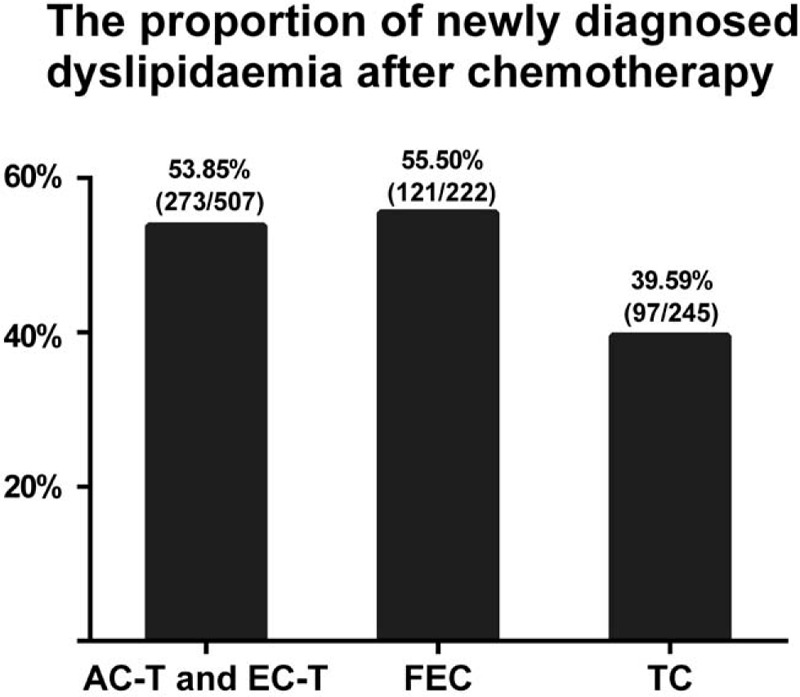
The percentage of newly diagnosed dyslipidemia after chemotherapy in AC-T and EC-T group, TC group, and FEC group.

Among the 1934 patients, there were 974 patients with normal serum lipids before chemotherapy. As many patients had increased serum lipid levels after chemotherapy, we further assessed the incidence of newly diagnosed dyslipidemia after chemotherapy (Figs. 5 and 6). Among the 974 patients with normal serum lipid levels before chemotherapy, there were total 491 patients (50.41%) newly diagnosed with dyslipidemia after chemotherapy. Stratified by menopausal status, the incidence of newly diagnosed dyslipidemia was higher in the postmenopausal group (56.14%) than that of the premenopausal (48.04%) group (*P* *=* .021). Then, stratified by chemotherapy regimens, the incidence of newly diagnosed hyperlipidemia was lower in FEC regimen (39.59%) than that of TC regimen (55.5%) and AC-T and EC-T regimens (53.85%; *P* < .001).

**Figure 7:**
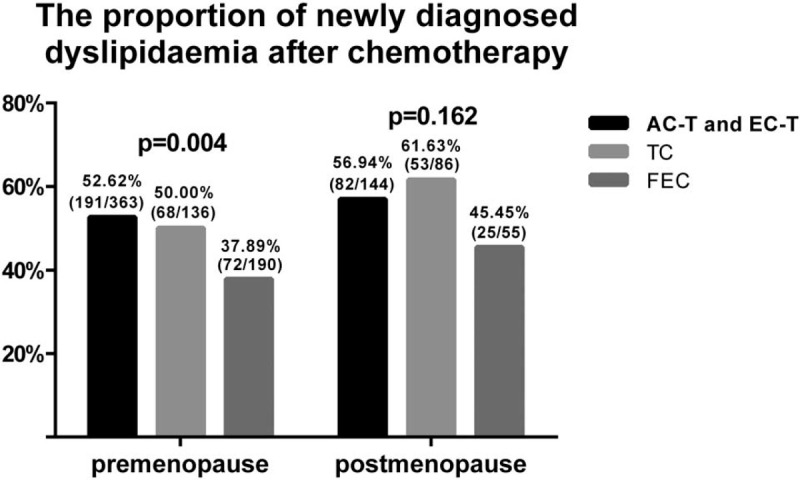
The percentage of newly diagnosed dyslipidemia after chemotherapy in AC-T and EC-T group, TC group, and FEC group in pre- and postmenopausal groups, respectively.

Moreover, we further layered participants according to chemotherapy regimens after stratifying them by menopausal status. Data can be seen in Figure 7. For the premenopausal, the incidence of newly diagnosed dyslipidemia after chemotherapy was also lower in FEC regimen (37.89%) than that of TC regimen (50%) and AC-T and EC-T regimens (52.62%; *P* *=* .004). However, for the postmenopausal, there was no significant difference in the incidence of newly diagnosed dyslipidemia after chemotherapy in the 3 chemotherapy regimens (*P* *=* .162).

### 3.5 Factors related to dyslipidemia after chemotherapy

A total of 974 patients had normal lipid levels before chemotherapy, and among these individuals, there were 491 new cases (50.41%) of dyslipidemia post-chemotherapy. To explore the potential clinicopathological parameters associated with newly diagnosed dyslipidemia after chemotherapy, we performed univariate and multivariate analyses (Table 6 ). With respect to TG levels, the univariate analysis showed that post-chemotherapy elevated TG levels were statistically associated with age between 40 and 50 years old, mastectomy, chemotherapy regimens containing taxane (TC regimen, AC-T, and EC-T regimens) and BMI > 24 (*P* < .05). By incorporating these factors into the logistic regression model, we found that BMI > 24, mastectomy and chemotherapy regimens containing taxane were still independent predictors of high TG levels after chemotherapy (*P* < .05). For TC levels, univariate analysis showed that post-chemotherapy elevated TC levels were statistically associated with postmenopausal status, age > 40 years old, BMI > 24, ER negative, preoperative hypertension, and anthracycline-plus-taxane-based regimens (AC-T and EC-T) (*P* < .05). After adjusting for multiple baseline covariates, only BMI > 24 was independent predictor of high TC levels after chemotherapy (*P* < .05). In terms of HDL-C, both univariate and multivariate analysis showed that BMI > 24 and anthracycline-plus-taxane-based regimens (AC-T and EC-T) were independent predictors of low HDL-C levels after chemotherapy (*P* < .05). With respect to LDL-C levels, univariate analysis showed that post-chemotherapy elevated LDL-C levels were statistically associated with BMI > 24, ER negative, age > 40 years old, and postmenopausal status (*P* < .05). After adjusting for multiple baseline covariates, we found that age between 40 and 50 years, BMI > 24 and ER negative status were still independent predictors of high LDL-C levels.

**Table 6:**
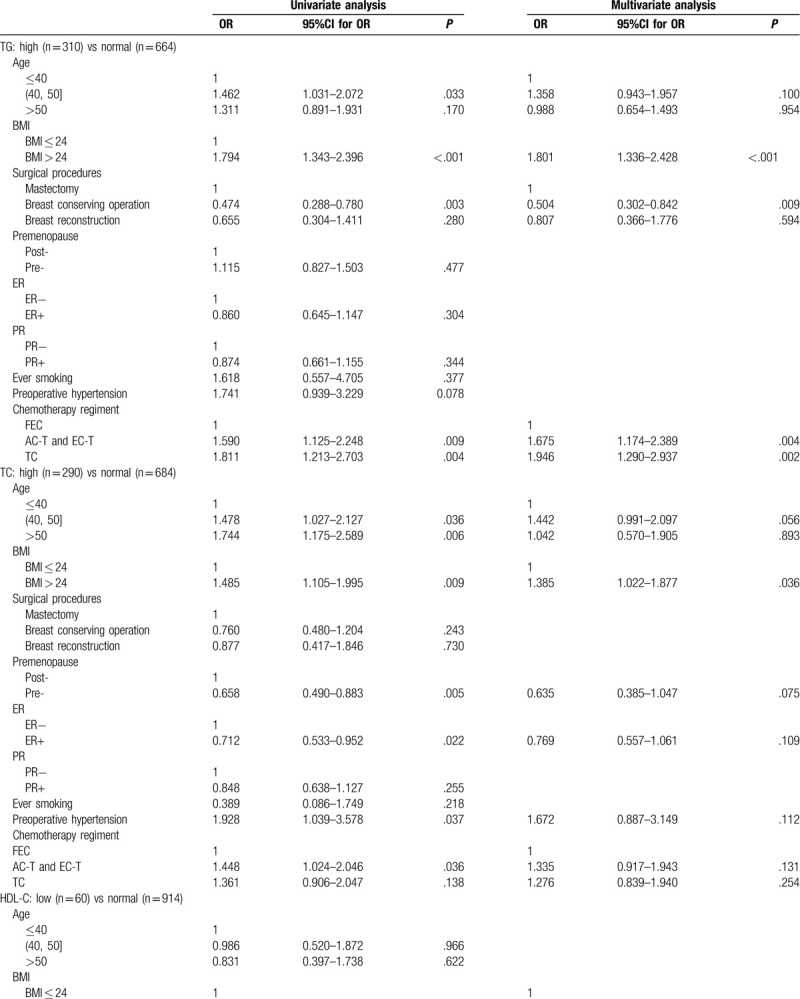
Factors associated with dyslipidemia based on univariate and multivariate analysis, by stepwise logistic regression.

**Table 6 (Continued):**
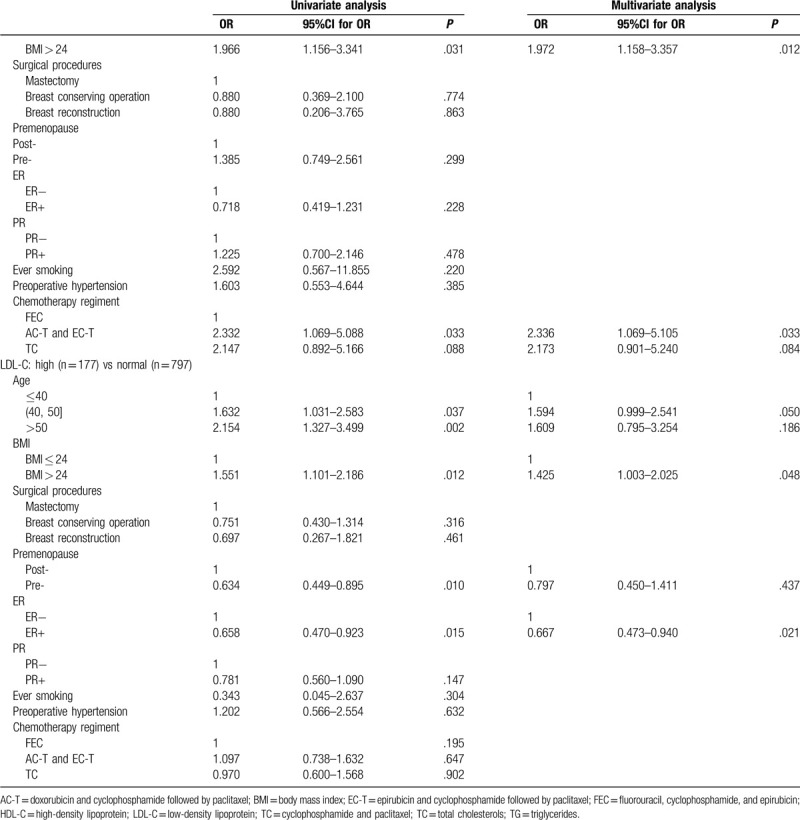
Factors associated with dyslipidemia based on univariate and multivariate analysis, by stepwise logistic regression.

## 4 Discussion

This is one of the first retrospective cohort studies to investigate the status of serum lipids pre- and post-chemotherapy, as well as to compare lipid level alterations among different chemotherapy regimens. The levels of TG, TC, and LDL-C were significantly elevated in patients who received chemotherapy. With respect to different regimens, FEC regimen had less side effects on lipid profiles and the incidence of newly dyslipidemia after chemotherapy was also lowest in patients who received FEC than that of the patients received other regimens. Moreover, after adjusting for multiple baseline covariates, anthracycline-plus-taxane-based regimens (AC-T and EC-T) were still statistically associated with high levels of TG and low levels of HDL-C after chemotherapy. Additionally, the magnitude of the alterations in lipid profiles (TG, TC, HDL-C, and LDL-C) was greater in premenopausal patients than that of the postmenopausal patients.

A few studies have addressed the side effects of chemotherapy regimens on lipid levels in postoperative BC patients. As described previously, an increase in cholesterol (TC) was observed in patients with early BC who received chemotherapy only.^[20]^ Li et al reported that the levels of TC, TG, LDL-C, and apolipoprotein B were significantly higher among post-chemotherapy patients than among pre-chemotherapy patients, but HDL-C levels were the opposite.^[21]^ Similarly, Dieli et al found that after BC patients receiving chemotherapy, their TG, TC, and LDL-C significantly increased; while HDL-C was decreased.^[22]^ Consistent with previous research results, our findings showed that the levels of TG, TC, and LDL-C were significantly elevated in patients who received chemotherapy. A possible explanation for the elevated lipid profiles may be that chemotherapy may directly cause endothelial dysfunction and insulin resistance, leading to cytokine alterations, finally increasing serum lipid profiles.^[23–25]^ Previous study had reported that children long term exposed to anthracycline treatment exhibited a marked preclinical vasculopathy, characterized by endothelial dysfunction and increased arterial stiffness, resulting in a deteriorated cardiovascular function.^[26]^ Another reason why serum lipids are elevated after chemotherapy may be that some chemotherapy regimens may enhance systemic oxidative stress to cause lipid peroxidation, thus, resulting in toxicity to the liver,^[27]^ which is a vital organ that regulates lipid metabolism.^[28]^ Previously, Monika et al found that doxorubicin reduces the expression of ABCA1 and apoA1 in HepG2 cells and the expression of ABCA1 in liver cells is a major contributor to HDL-C levels via its role in cholesterol efflux.^[29]^ Besides anthracycline, the side effects of taxane on serum lipids were also reported. Panis et al reported that paclitaxel treatment significantly reduced plasma HDL-C levels and increased plasma hydroperoxide levels when compared to BC patients without chemotherapy.^[30]^ Also, another previous study^[31]^ showed that the cardiotoxicity of the taxane-based regimens has been worked by enhancing the additive effect of doxorubicin-induced cardiomyopathy.

It is well known that AC-T, EC-T, TC, and FEC are common chemotherapy regimens for postoperative BC patients. And in this study, we found that the patients’ lipid profiles almost tended to a worse situation no matter which regimen they received. Whereas, the magnitude of alterations of lipid profiles varied in different regimens. Previous studies have demonstrated that glucocorticoids are closely connected to lipid levels.^[32–34]^ Some apparent unfavorable changes have been reported in regard to LDL-C and vLDL-C metabolism with the utilization of glucocorticoids by Ross and Marais.^[35]^ Dexamethasone, as a routine premedication, is given to BC patients who received paclitaxel to avoid hypersensitivity reactions and vomiting.^[36]^ In the present study, we demonstrated that FEC regimen had less side effects on lipid profiles and the incidence of newly dyslipidemia after chemotherapy was also lowest in patients receiving FEC than that of the other chemotherapy regimens. This may be explained by the utilization of paclitaxel and dexamethasone premedication during chemotherapy.^[37]^

Zagar et al demonstrated that a significant proportion of patients had dyslipidemia after chemotherapy.^[12]^ In addition, Yeo et al^[38]^ reported that older age, postmenopausal status, weight gain or overweight/obese status, and having received taxane-based regimens and corticosteroid premedication during adjuvant chemotherapy were related to chemotherapy-induced dyslipidemia. Similarly, in our study, we explored the risk factors related to high levels of TG, TC, and LDL-C and low levels of HDL-C in patients with normal lipid profiles before chemotherapy. After adjusting for multiple factors, we found that BMI > 24 was an independent risk factor for high levels of TG, TC, LDL-C and low level of HDL-C after chemotherapy. In accordance with our results, previous studies have confirmed that BMI was positively correlated with serum lipids.^[39]^ Similarly, Pan et al demonstrated that BC patients were more likely to achieve dyslipidemias when becoming fatter.^[40]^ Additionally, our results also showed that anthracycline-plus-taxane-based regimens were still statistically associated with a high level of TG and a low level of HDL-C after chemotherapy compared with FEC regimen. A possible explanation may be that the liver toxicity caused by chemotherapy varied in different regimens, and thus, the alterations in lipid profiles were different among the different chemotherapy regimens. Compared with FEC regimen, AC-T and EC-T regimens contain an extra chemotherapeutic drugs – —Taxanes, which may have side effects on lipid profiles or may enhance the side effects of anthracycline on serum lipids.^[30]^ As well as, AC-T and EC-T usually contain 2 more chemotherapy cycles than FEC regimen, thus, the cumulative effects of chemotherapy may be more remarkable in anthracycline-plus-taxane-based regimens. A surprising finding was that estrogen receptor negative status was an independent risk factor for high LDL-C levels. In contrast to earlier findings, for instance, Efstathiadou reported that estrogen receptor β was associated with high LDL-C levels in men independently of confounders.^[41]^ Notably, the subjects in our study were all women, and it is possible that estrogen receptors in women have the benefit of higher estrogenic exposure throughout their reproductive years.^[42]^ Thus, more studies are urgently needed to confirm whether the ER status is associated with high LDL-C levels.

Li et al^[43]^ reported that serum lipid profiles increased significantly after hysterectomy, and the magnitude of alterations was more remarkable in younger women. Similarly, Tian et al^[44]^ also reported that the younger group showed a greater increase in TC and LDL-C levels during chemotherapy than the 41–65-year-old group. Consistent with their findings, in our study, it is worth noting that the magnitude of lipid profiles alterations was more prominent in premenopausal patients than that of the postmenopausal group. These results may be attributed to chemotherapy-induced ovarian failure, which results in elevated serum lipid levels due to the lack of estrogen.^[45–47]^ Moreover, it is not difficult to understand that postmenopausal ovarian function is poor; thus, external interference has limited effects on failed ovary. Whereas, premenopausal BC patients have higher levels of sex hormones and better lipid metabolism status, thus, lipid profiles are more sensitive to chemotherapy regimens. A part from these results, a seemingly contradictory result was that the incidence of newly diagnosed dyslipidemia after chemotherapy was higher in the postmenopausal group (56.14%) than that of the premenopausal (48.04%) group. It was due to the high baseline levels of lipid profiles in postmenopausal BC patients.

To the best of our knowledge, the present study was one of the first to provide comprehensive information on the side effects of chemotherapy on serum lipid levels and to explore the effects of different chemotherapy regimens on lipid profiles in both premenopausal and postmenopausal BC patients. Moreover, factors potentially implicated in newly diagnosed dyslipidemia after chemotherapy were also identified in a relatively large sample. However, there are still several limitations to this study that should be highlighted. First, as a retrospective study, some clinicopathological data were lost and some confounding factors could not be completely excluded. Secondly, the present study lacks data on calorie intake and consumption, energy variation and body composition, such as dietary intake, physical activity, and basic metabolic rates. Recently, several studies showed that BC survivors treated with chemotherapy mainly experienced weight loss rather than weight gain.^[48,49]^ Wang et al and Irwin et al reported that the mean weight change was in fact a loss of 0.34 and 0.4 kg, respectively.^[50,51]^ These studies indicated that the increase in dyslipidemia may be more likely to be associated with chemotherapy rather than weight again. Additionally, the mechanism of lipid profiles increase is not yet clear, and many factors may also have intertwining effects on lipids, thus the specific mechanism of chemotherapy-associated lipid profiles alterations was not explained clearly. Last but not least, we lacked the prognostic value of follow-up in later stage and we did not further explore the relationship between dyslipidemia and CVD and the long-term prognosis of BC patients. Thus, further studies are urgently warranted to supplement and confirm the relationship between chemotherapy and blood lipid metabolism.

## 5 Conclusions

In summary, adjuvant chemotherapy may elevate lipid profiles for postoperative BC patients, especially for premenopausal women. In addition, the effects of chemotherapy on lipid profiles vary based on different chemotherapy regimens and anthracycline-based regimen had less side effects on lipid profiles compared with regimens containing taxane. Therefore, lipid monitoring and dyslipidemia prevention and treatment should be taken into consideration during chemotherapy.

## Acknowledgments

The authors gratefully acknowledge the whole staff of the Department of Breast Surgery, West China Hospital, who generously provided assistance in the collection of data throughout the duration of the study.

## Author contributions

All authors contributed to the conception, design and interpretation of the data; the preparation of the manuscript; and the final editing and approval of the manuscript. All authors read and approved the final manuscript.
